# Mapping the Global Network of Extracellular Protease Regulation in Staphylococcus aureus

**DOI:** 10.1128/mSphere.00676-19

**Published:** 2019-10-23

**Authors:** Brittney D. Gimza, Maria I. Larias, Bridget G. Budny, Lindsey N. Shaw

**Affiliations:** a Department of Cell Biology, Microbiology & Molecular Biology, University of South Florida, Tampa, Florida, USA; University of Nebraska Medical Center

**Keywords:** *Staphylococcus aureus*, gene regulation, proteases, transcriptional regulation, virulence factors

## Abstract

A primary function of the extracellular proteases of Staphylococcus aureus is to control the progression of infection by selectively modulating the stability of virulence factors. Consequently, a regulatory network exists to titrate protease abundance/activity to influence the accumulation, or lack thereof, of individual virulence factors. Herein, we comprehensively map this system, exploring the regulation of the four protease loci by known and novel factors. In so doing, we determined that seven major elements (SarS, SarR, Rot, MgrA, CodY, SaeR, and SarA) form the primary network of control, with the latter three being the most powerful. We note that expression of aureolysin is largely repressed by these factors, while the *spl* operon is subject to the strongest upregulation of any protease loci, particularly by SarR and SaeR. Furthermore, when exploring *scpA* expression, we find it to be profoundly influenced in opposing fashions by SarA (repressor) and SarR (activator). We also present the screening of >100 regulator mutants of S. aureus, identifying 7 additional factors (ArgR2, AtlR, MntR, Rex, XdrA, Rbf, and SarU) that form a secondary circuit of protease control. Primarily, these elements serve as activators, although we reveal XdrA as a new repressor of protease expression. With the exception or ArgR2, each of the new effectors appears to work through the primary network of regulation to influence protease production. Collectively, we present a comprehensive regulatory circuit that emphasizes the complexity of protease regulation and suggest that its existence speaks to the importance of these enzymes to S. aureus physiology and pathogenic potential.

**IMPORTANCE** The complex regulatory role of the proteases necessitates very tight coordination and control of their expression. While this process has been well studied, a major oversight has been the consideration of proteases as a single entity rather than as 10 enzymes produced from four different promoters. As such, in this study, we comprehensively characterized the regulation of each protease promoter, discovering vast differences in the way each protease operon is controlled. Additionally, we broaden the picture of protease regulation using a global screen to identify novel loci controlling protease activity, uncovering a cadre of new effectors of protease expression. The impact of these elements on the activity of proteases and known regulators was characterized by producing a comprehensive regulatory circuit that emphasizes the complexity of protease regulation in Staphylococcus aureus.

## Abstract (precis)

The complex regulatory role of the proteases necessitates very tight coordination and control of their expression. While this process has been well studied, a major oversight has been the consideration of proteases as a single entity rather than as 10 enzymes produced from four different promoters. As such, in this study, we comprehensively characterized the regulation of each protease promoter, discovering vast differences in the way each protease operon is controlled. Additionally, we broaden the picture of protease regulation using a global screen to identify novel loci controlling protease activity, uncovering a cadre of new effectors of protease expression. The impact of these elements on the activity of proteases and known regulators was characterized by producing a comprehensive regulatory circuit that emphasizes the complexity of protease regulation in Staphylococcus aureus.

## INTRODUCTION

Staphylococcus aureus is an opportunistic human pathogen known for causing both hospital- and community-acquired infections. It is capable of causing a plethora of diseases that range from minor skin and soft tissue infections, such as boils and carbuncles, to septicemia, endocarditis, osteomyelitis, and toxic shock syndrome (1–3). This broad disease potential can be attributed to the coordinated production of a wealth of virulence factors by S. aureus within the human host. Collectively, these elements allow the pathogen to evade phagocytosis, promote abscess formation, travel from initial sites of infection to invade new tissues, and induce a variety of syndromes (4). These virulence-causing entities can be divided into two broad groups: adherence factors and exoproteins. Adherence factors are responsible for the attachment of S. aureus to host tissues so that colonization may occur (5) and can also interfere with the host immune system to facilitate immune evasion (6). Conversely, exoproteins are secreted by S. aureus and function to acquire nutrients by breaking down host tissues and, more importantly, target the immune system, engendering immune subversion (7).

**FIG 1:**
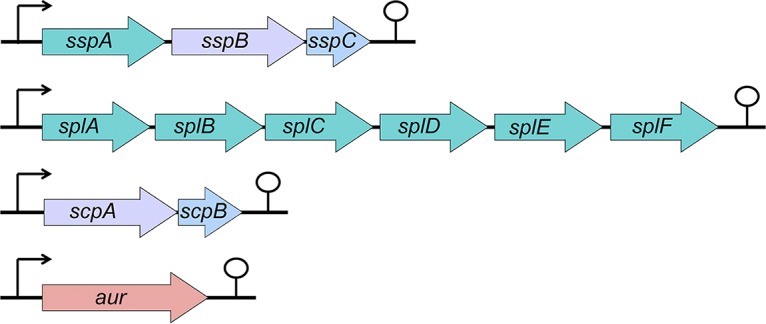
Genetic organization of the S. aureus secreted protease loci. The colors of arrows are representative of catalytic activity classification: metalloprotease in pink; serine proteases in green; cysteine proteases in purple; and the inhibitors of the staphopains (the staphostatins) in blue.

Parts of this cadre of secreted factors are 10 extracellular proteases, which are produced by almost every S. aureus strain (Fig. 1) (8, 9). These include the following: a metalloprotease, aureolysin (*aur*); a serine protease, V8 (*sspA*); two cysteine proteases, staphopain B (*sspB*) and staphopain A (*scpA*); and six serine protease-like enzymes (*splABCDEF*) (9, 10). The functions of these enzymes have been studied by ourselves and others and include their ability to hydrolyze a variety of host proteins as well as self-derived toxins. With regard to host factors, the secreted proteases have been demonstrated to proteolyze proteins such as fibrinogen, elastin, and the heavy chains of immunoglobulins to promote tissue invasion, immune system evasion, and the dissemination of infection (11–13). In the context of the self-degradome, these enzymes can cleave multiple virulence determinants to promote bacterial invasion, immune evasion, and survival. For example, aureolysin was shown to control the stability of both phenol-soluble modulins and alpha-toxin (14, 15) as well as the adhesin clumping factor B (ClfB) (16), while SspA is able to cleave surface protein A (SpA) and the fibrinogen-binding proteins (FnBPs) (17, 18).

Recently, our group assessed the importance of secreted proteases in S. aureus pathogenesis using a strain where all 10 enzymes were deleted (19). Here, we demonstrated that secreted proteases are required for growth in whole human blood, serum, peptide-rich medium, and in the presence of antimicrobial peptides. Additionally, these enzymes are also necessary for S. aureus to resist phagocytosis by human granulocytes and monocytes. Most striking, however, were the *in vivo* phenotypes of this mutant, where a decrease in dissemination and abscess formation were observed in infected mice compared to in the wild type. Conversely, when assessing mortality, the complete protease-null strain demonstrated pronounced hypervirulence. These contrasting phenotypes were explained using proteomics, where an increase in the stability of secreted and surface-associated virulence factors was demonstrated *en masse* in the mutant, thus facilitating more aggressive and deadly infections. Importantly, many of these findings were also demonstrated in a companion study by Zielinska et al. (20). As such, it would appear that secreted proteases have a biphasic role in infection, serving on the one hand to modulate the stability of self-derived pathogenic determinants, so as to control disease severity and progression, while at the same time playing their own direct role by cleaving host proteins to promote invasion, immune evasion, and survival.

Given the complex regulatory role of S. aureus proteases during infection, it follows that there must be, and indeed is, tight control of their expression mediated by a collection of different factors. This is evidenced by the number of elements that have been identified thus far as influencing protease production, including RNAIII SarS, SarR, SarA, SarV, SarX, SarZ, ArlRS, CodY, Rot, MgrA, and SaeRS (21–33). Of these factors, SarS, SarR, CodY, Rot, MgrA, SaeR, and SarA are considered the primary regulators, with each being shown to directly influence protease transcription (21–27). A major oversight when studying the control of protease production in S. aureus, however, has been the consideration of these factors as a single entity rather than as 10 enzymes produced from four different promoters. Of the seven major regulators, only SarA and Rot have been explored in the context of all four protease promoters (9, 10), with SarA shown to specifically repress the transcription of *aur*, *scpA*, and *ssp* but not *spl* (9, 10), while Rot has been described as a direct negative regulator of all secreted protease operons (23). For the other primary regulators, CodY has been shown to directly repress *ssp* transcription (22), while SarS and SarR have been explored only in the context of *aur* and *ssp* promoter binding (21). Finally, MgrA has been shown to activate *aur*, *ssp*, and *spl* transcription (25, 34), while SaeR has been described as an activator for *spl* but a repressor for *aur* (24).

Consequently, the overarching goal of this study was to explore and further our understanding of the regulation of secreted proteases by known regulatory factors in S. aureus while concurrently uncovering new effectors of protease transcription. Accordingly, we present a comprehensive mapping of protease regulation by all known S. aureus transcription factors in community-acquired methicillin-resistant S. aureus (CA-MRSA) strain USA300.

## RESULTS AND DISCUSSION

### Exploring the differential regulation of protease expression by primary regulators.

To date, seven different transcriptional regulators (Rot, CodY, SarA, SarS, MgrA, SarR, and SaeR) (21–27) have been identified as being the primary modulators of secreted protease expression. An oversight, however, is the consideration of S. aureus proteases as a single entity rather than as 10 enzymes produced from four distinct loci (Fig. 1). Thus, although these elements do indeed have the capacity to regulate the expression of one or more proteases, only a few have been explored in the context of all four operons. Therefore, our initial goal was to fill in missing gaps using quantitative real-time PCR (qRT-PCR). To assess this, wild-type and regulator mutant strains were grown to postexponential phase (5 h), which is a known window of peak protease expression (9), and assessed for the expression of each protease operon.

**FIG 2:**
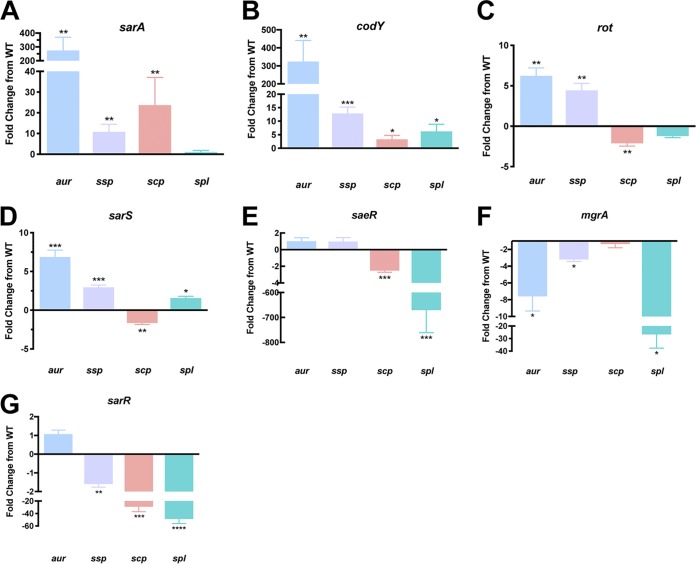
Individual protease loci are differentially controlled by major regulators of S. aureus. qRT-PCR was used to determine transcript levels for *aur*, *scp*, *ssp*, and *spl* in regulator mutants after 5 h of growth. The strains used were wild type (WT) USA300 Houston (HOU) and mutants for *sarA* (A), *codY* (B), *rot* (C), *sarS* (D), *saeR* (E), *mgrA* (F), and *sarR* (G). RNA was isolated from three independent cultures. The 16S rRNA gene was used as an internal control. Fold change from WT was determined using the 2^−ΔΔ^*^CT^* method. Student’s *t* tests were used to determine statistical significance. *, *P* < 0.05; **, *P* < 0.01; ***, *P* < 0.001; ****, *P* < 0.0001 relative to the wild-type strain. Error bars show the standard deviations (SDs).

We began with the best-studied regulator, SarA, whose ability to repress the transcription of *aur*, *scpA*, and *ssp* but not *spl* has been well established (9, 10). Here, our analysis provided the expected results: in the absence of SarA, there was a 275-fold increase in *aur*, 10.9-fold increase in *sspA*, and a 23.7-fold increase in *scpA* transcript levels, with no changes in *spl* expression (Fig. 2A).

Next, we investigated CodY, whose ability to influence protease expression was identified by microarray analysis in UAMS-1 (22). There, Majerczyk et al. (22) found that in the absence of CodY, *sspA* had increased transcript levels. Additionally, in the same study, CodY was shown to bind the *spl*, *sspA*, and *aur* promoters; however, the binding to *aur* and *spl* was deemed biologically irrelevant, as changes in their expression were not observed upon *codY* deletion. As such, the ability of CodY to modulate expression of *aur*, *scpA*, and *spl* has not been previously described. Herein, in the absence of CodY, we observed a significant 324-fold increase in *aur*, 12.8-fold increase in *sspA*, 3.3-fold increase in *scpA*, and 6.2-fold increase in *spl* transcript levels (Fig. 2B). Collectively, these data suggest that CodY is a negative regulator of secreted protease expression that rivals SarA in its potency.

We next considered Rot, which was first shown to negatively regulate *sspA* and *spl* transcription in a RN6390 microarray (35). In another study assessing *aur* and *sspA* regulation in strain 8325-4, Rot functioned as a direct repressor of both loci (25). In support of these studies, others have demonstrated that Rot represses *aur* and *sspA* while also directly repressing *spl* through promoter binding in strain LAC (23). Additionally, in the same study, Rot was shown for the first time to directly repress *scpA* transcription. In our study, upon *rot* inactivation, there were significant increases of 6.2-fold for *aur* and 4.5-fold for *sspA* transcript levels, which is in line with previous research (23). Additionally, a significant 2.1-fold decrease in *scpA* expression along with no change for *spl* was observed, contradicting previous studies, where increased transcription for both was observed upon *rot* deletion (Fig. 2C). We note, however, that previous studies regarding Rot regulation differ from ours through the use of medium supplemented with different nutrients. Specifically, in work by Mootz et al. (23), growth medium was supplemented with glucose, which has been documented as repressing the *agr* quorum sensing system via the decreased pH produced from carbon metabolism (23, 36, 37). As such, this decrease in *agr* activity could alter the expression of downstream factors also capable of regulating the secreted proteases. Similarly, Said-Salim et al. (35) used Casamino Acids-yeast extract-glycerol phosphate broth for their studies. Here, the addition of glycerol, as well as the use of an entirely different complex medium, altered the activity of other transcriptional regulators such as CodY, CcpE, CcpA, and RpiRC, which are known to sense the carbon status of the cell (38). Therefore, while Rot has the potential to regulate all four protease loci, our data suggest that Rot primarily controls expression of *aur* and *sspAB*, likely in an *agr*-dependent manner.

SarS was formerly shown to have no significant effect on *aur* and *ssp* transcription during investigation in strain 8325-4 (27). Oscarsson et al., however, established that when *sarS* is overexpressed in 8325-4, *aur* and *sspA* transcription is suppressed (25). In support of a role in *sspA* regulation, another study showed that SarS could bind the *sspA* promoter (27). To date, the effects of SarS on *scpA* and *spl* transcription have not yet been investigated. Our analysis of protease transcription in the absence of SarS revealed significant increases for *aur* (6.9-fold), *sspA* (2.9-fold), and *spl* (1.6-fold) but a 1.7-fold decrease in *scpA* transcript levels (Fig. 2D). These data thus support a role for SarS as a repressor of *aur* and *sspA* expression and identify the *spl* operon as a new target of negative regulation by this factor. Conversely, we reveal *scpA* as a being activated by SarS, demonstrating, as with our data for Rot, that each of the four proteases are often subject to differential and opposing regulation by the same element.

The ability of SaeR to influence protease expression was previously described by microarray analysis, where, in the absence of SaeR/S in strain LAC, there was a decrease in *spl* transcription (24). Furthermore, in that same study, it was observed that this effect was direct, as SaeR was shown to bind to the *spl* promoter. Additionally, in the same background, Cassat et al. showed a decrease in SplA-F protein levels following *sae* inactivation (39). In support of this, we observed a striking 671-fold decrease in *spl* transcript levels upon *saeR* deletion, which is the most pronounced alteration in expression for any protease observed in this study (Fig. 2E). With regard to *aur*, the previously referenced studies revealed an increase in *aur* transcription (24) as well as an increase in Aur protein levels (39) in the absence of *saeRS*. In our study, however, no change in transcription was observed, which is in line with Oscarsson et al., who derived similar findings in strain RN6390 (25). Of note, the changes observed during microarray and proteomic analyses were during stationary phase rather than postexponential phase. Therefore, the disagreement regarding *aur* regulation could be a product of different time points used for assessment. This is supported by our observation that, when analyzed throughout growth, SaeRS is the only major regulator in S. aureus to demonstrate a rebound in transcriptional activity during stationary phase (our unpublished observation). This suggests that SaeRS may have various or biphasic functions with regard to virulence factor regulation during S. aureus growth. Regarding *scpA*, the effect of SaeR on transcription has not until now been investigated. Herein, we observed a 2.5-fold decrease in *scpA* transcription in the absence of SaeR, indicating that, similarly to the *spl*s, it is activated by this factor. Lastly, no change in *sspA* transcription was observed, which, while in line with Oscarsson et al. (25), contradicts Cassat et al. (39), who observed an increase in SspA and SspB protein levels during stationary phase. As previously suggested, this conflict is likely explained by the varying impact of SaeRS during different growth phases. As such, our data support a role for SaeR during postexponential growth in the activation of *spl* and identify *scpA* as a new target for SaeR upregulation.

We next investigated MgrA, which was previously shown to activate *aur* and *sspA* transcription in 8325-4 (25). Using RNA sequencing in LAC, others have shown that the absence of MgrA decreased *aur* and *spl* transcript levels (34). Herein, in agreement with previous studies, we observed a significant 7.6-fold decrease in *aur*, 3.2-fold decrease in *sspA*, and 26.7-fold decrease in *spl* transcript levels (Fig. 2F). Lastly, until now, the effect of MgrA on *scpA* had not been investigated. In our study, no changes in *scpA* transcript levels were identified, which again demonstrates differential regulation of the various protease loci. This is particularly interesting, as it is an additional example of the two staphopain enzymes (SspB and ScpA), which share strong homology (40–42) although quite different substrate specificities (42), as being regulated in opposing fashions.

Finally, we investigated SarR, which was formerly shown to positively affect *aur* and *sspA* transcript levels in 8325-4 (21). In contrast, in another study, it was shown to negatively affect *aur* when overexpressed in an 8325-4 *agr sarA* double mutant (25). Interestingly, however, in our study, no change in *aur* transcript levels was detected in the absence of *sarR*. When considering *ssp* expression, we observed a significant 1.6-fold decrease in transcript levels (Fig. 2G) in the *sarR* mutant, which is in agreement with Gustafsson et al. (21). With regard to *scpA* and *spl*, SarR was not previously investigated as controlling their transcription. Herein, we observed a significant 29.1-fold decrease in *scpA* and 48.8-fold decrease in *spl* transcript levels. Our data therefore support a role for SarR in upregulating the *ssp* operon to a minor extent while serving as one of the strongest activators of *scpA* and *spl* expression identified thus far.

### Defining the pathway of control for secreted protease expression by known major regulators.

**FIG 3:**
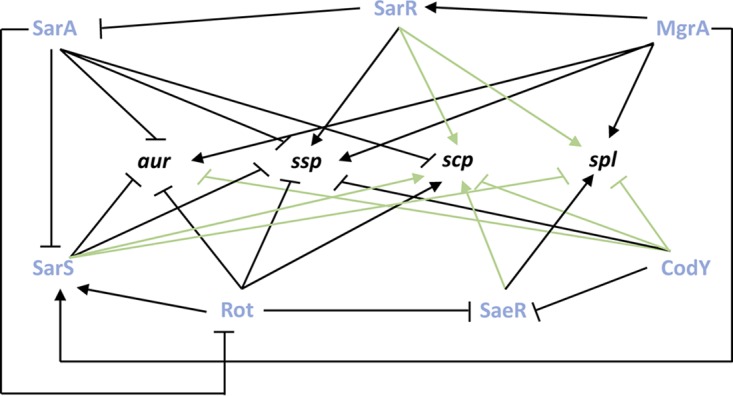
Primary network of control for individual protease loci. Shown are transcriptional regulation events for the seven primary protease regulators of S. aureus on the four individual protease loci. Bars indicate repression, and arrows represent activation. New regulatory pathways identified herein between the primary regulators and the protease loci are shown in green.

Collectively, our findings confirm 14 regulatory pathways for secreted protease transcription while identifying eight new nodes of expression (Fig. 3). For *aur*, we found it was regulated by CodY in addition to SarA, Rot, SarS, and MgrA. Interestingly, with the exception of MgrA, each of these factors engenders repression of *aur* expression, with some (SarA and CodY) exerting profound influence. This is perhaps explained by the observation that aureolysin sits atop the protease activation cascade, which flows from Aur to V8 and then staphopain B (11, 43–45). As such, repressing aureolysin would allow the S. aureus cell to keep the majority of proteases’ activity restrained by the single act of limiting expression from P*_aur_*. This would be to the cells advantage as, although proteases are undoubtedly valuable enzymes with important roles, they are also destructive in nature. Thus, limiting their activity until it is absolutely required is a major goal of living cells from all kingdoms (46, 47). This would be particularly true of aureolysin, given that it has among the broadest substrate specificities of any S. aureus protease (48). In the context of enzymes from the *ssp* operon, we did not identify new regulatory nodes but confirmed their broad regulation, albeit at modest levels, in a fashion that closely resembles that of *aur* control. This finding is again logical, given that the enzymes produced from these loci are part of the protease activation cascade referenced above.

Interestingly, much of the new knowledge generated herein involves the regulation of the more underappreciated proteases, staphopain A and the Spls. While the importance of *scpA* in virulence has been shown through *in vivo* studies, as well as by its ability to cleave specific host proteins (13, 49, 50), its transcriptional regulation has been underexplored. While it has been shown previously that *scpA* is regulated by Rot and SarA, we identified herein that SarS, CodY, SaeR, and SarR also control its expression. While much of this regulation is at modest levels, *scpA* expression is profoundly influenced in opposing fashions by SarA (repressor) and SarR (activator). This presents a scenario whereby the presence of this enzyme during infection could be discretely titrated, with high SarA activity resulting in decreased staphopain A, while elevated SarR levels would engender significant production of this enzyme. This could then provide rapid niche-specific control of the pathogenic process through staphopain A activity (or lack thereof) toward self- and host-derived proteins. The need for such a network of opposing and stringent control is supported by the observation that staphopain A is one of only two S. aureus secreted proteases with a broad and promiscuous substrate specificity (aureolysin being the other) (51); thus, tightly modulating its influence is a necessity for a coordinated and controlled infectious process.

When exploring control of *spl* expression, we note that this operon is subject to some of the strongest regulation observed for any protease loci in this study. Specifically, MgrA, SarR, and SaeR each bring about profound upregulation of the *spl* operon, to levels that rival and, in the case of SaeR, exceed, that of SarA and CodY for protease control. This is of interest because the Spls are well known for their narrow substrate specificity (52–54). Indeed, these enzymes share strong homology and many enzymatic characteristics with the exfoliative toxins of S. aureus. In the case of these latter proteases, they have only a single known target, desmoglein-1 in the skin of humans, the cleavage of which results in scalded skin syndrome (55). The Spl enzymes are projected to have a similarly narrow range of substrates (56); thus, it is logical that the cell would limit the production of these enzymes until it finds itself in an environment where their activity would prove beneficial. As such, it is logical that the presence and activity of the Spl enzymes can be selectively and rapidly stimulated by these regulatory factors in response to environmental cues within the host to facilitate infection.

### Identification of a cadre of new effectors of protease activity.

**FIG 4:**
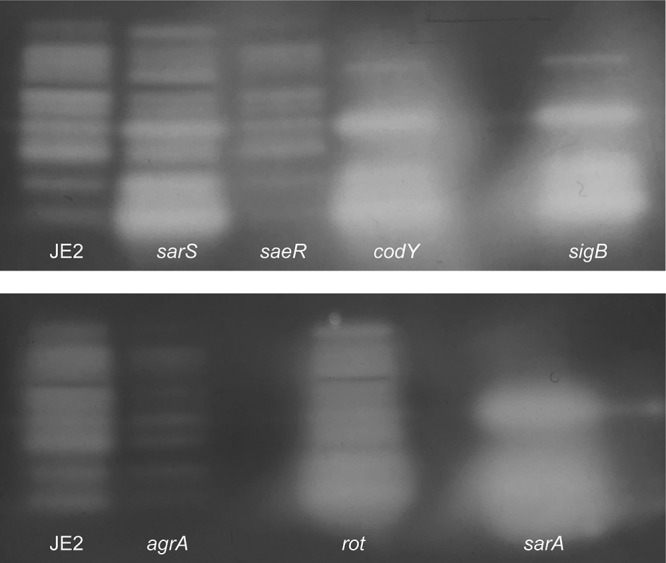
Impact of primary regulator mutation on protease activity. Gelatin zymography was performed to visualize protease activity on 15-h culture supernatants obtained from USA300 JE2 and mutants of *sarS*, *saeR*, *codY*, *sigB*, *agrA*, *rot*, and *sarA*. All strains were adjusted to equal optical densities prior to analysis.

**FIG 5:**
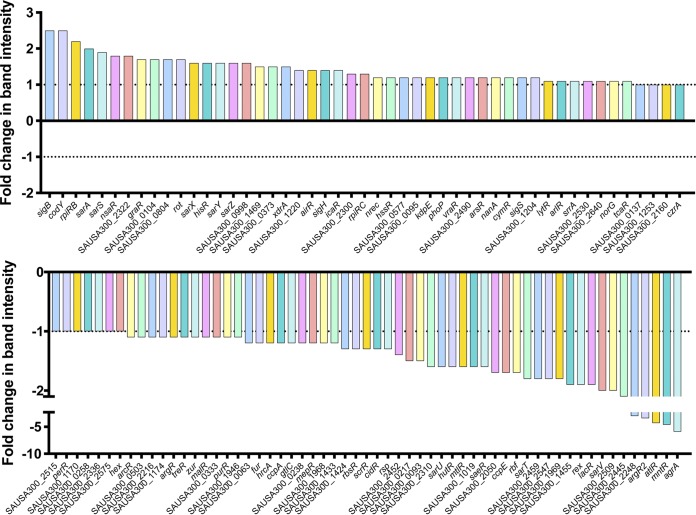
Quantitative profiling of protease activity for all available regulator mutants of S. aureus. Zymogram band intensities from all 108 regulator mutants contained within the NTML were measured using densitometry (ImageJ). Depicted is fold change of band intensity relative to that of the USA300 JE2 wild-type strain.

Given the complex regulatory function of S. aureus secreted proteases, tight modulation of their expression is required. As such, we set out to more deeply characterize their network of control by uncovering novel effectors of their activity. This was achieved by screening all 108 available transcriptional regulator mutants within the Nebraska Transposon Mutant Library (NTML) (57) for alterations in proteolytic capacity. Culture supernatants from all strains grown for 15 h (a window of peak accumulation for secreted proteases) were prepared and subjected to zymography using gelatin as a substrate, as described by us previously (9). Of the 108 mutants screened, five of the seven primary regulators (*sarS*, *saeR*, *rot*, *sarA*, and *codY* mutants) were included as controls (*sarR* and *mgrA* mutants are not present in the NTML), along with two other major regulators of protease production: *agrA* and *sigB*. As expected, an increase in proteolytic activity was observed with *sarS*, *rot*, *sarA*, *codY*, and *sigB* mutants, while a decrease was observed for *saeR* and *agrA* mutants, in comparison to that in the wild type (Fig. 4). For all strains, the intensities of proteolytic banding resulting from gelatin degradation were assessed visually and by densitometry using ImageJ software (Fig. 5).

Excluding the known major regulators, a total of 45 mutants were identified as having notable alterations in proteolytic activity from our screen, with 26 found to have decreased proteolysis (see Table S1 in the supplemental material), while 19 had an increase (Table S2). When assessing mutants that showed increased proteolysis, we identified SarX and NsaR, which were both previously identified as regulating proteases. SarX has been shown to repress *sspA* transcription in strain RN6390 (31), while NsaR was shown to be a repressor of *scpA*, *sspA*, and *splA-F* in strain SH1000 (58). When considering mutants that had decreased proteolysis, we noted SarV and CcpE, both of which have been implicated in modulating protease activity. Specifically, *sarV* disruption in RN6390 led to a decrease in transcription for *aur*, *scpA*, and *splA* (32), while loss of *ccpE* in strain Newman results in impaired expression of all protease loci (59).

10.1128/mSphere.00676-19.3 TABLE S1 Transcriptional regulators identified as producing a decrease in protease activity upon transposon disruption. *^a^* Strains chosen for further study are highlighted in grey. *^b^* NE#, NTML strain number. *^c^* N/A, gene name has not yet been assigned. *^d^* Transcriptional regulator family assignment is from reference 93. Download Table S1, PDF file, 0.03 MB. Copyright © 2019 Gimza et al. 2019 Gimza et al. This content is distributed under the terms of the Creative Commons Attribution 4.0 International license.

10.1128/mSphere.00676-19.4 TABLE S2 Transcriptional regulators identified as producing an increase in protease activity upon transposon disruption. *^a^* Strains chosen for further study are highlighted in grey. *^b^* NE#, NTML strain number. *^c^* N/A, gene name has not yet been assigned. *^d^* Transcriptional regulator family assignment is from reference 93. Download Table S2, PDF file, 0.02 MB. Copyright © 2019 Gimza et al. 2019 Gimza et al. This content is distributed under the terms of the Creative Commons Attribution 4.0 International license.

Beyond these known factors, we identified a number of intriguing regulators which have yet to be implicated in protease regulation. Of these, several displayed a prominent decrease in protease activity, including SarU. This regulator is an understudied transcription factor belonging to the Sar family, with many of its counterparts already known to have a role in regulating protease production (60). In addition, a notable decrease in protease activity was also observed for mutants of *rbf* and *atlR*, which encode regulators known to control biofilm formation (61–63). Further, Rex and MntR, both of which regulate different aspects of cellular metabolism, also caused pronounced decreases in protease activity upon ablation. We also observed a decrease in protease activity upon disruption of *argR2*, which is located within the arginine catabolism metabolic element (ACME) found in USA300 strains (64). Finally, XdrA/*xdrA*, which has a role in immune evasion via its involvement in the production of protein A (65), was found to produce a notable increase in protease activity upon disruption.

### Exploring protease control via a secondary network of regulation.

To more deeply explore the new protease regulatory factors identified herein, the seven referenced above were chosen for more detailed study. First, each mutation was transduced into a clean USA300 HOU background, and protease activity was continuously monitored throughout growth (see Fig. S1). In agreement with results from our zymography screen, a decrease in protease activity was observed at all time points for mutants of *argR2*, *mntR*, *atlR*, *rbf*, *sarU*, and *rex*, while the *xdrA* mutant demonstrated a minor decrease in protease activity at early times points, but produced the expected increase in proteolysis thereafter. To ensure that the changes observed were not the result of a simple growth defect, growth curves were performed for all strains, revealing no notable alterations compared to the growth of the wild type (see Fig. S2).

10.1128/mSphere.00676-19.1 FIG S1 Protease activity profiling of novel regulator mutants during growth. Gelatin zymography was performed on USA300 HOU WT and mutant strain culture supernatants obtained at the times specified. Culture supernatants were concentrated and ran on an SDS-PAGE gel containing 0.1% gelatin. Strains used are indicated on each gel. Download FIG S1, PDF file, 8.3 MB. Copyright © 2019 Gimza et al. 2019 Gimza et al. This content is distributed under the terms of the Creative Commons Attribution 4.0 International license.

10.1128/mSphere.00676-19.2 FIG S2 Growth analysis of novel protease regulator mutants. USA300 HOU WT and regulator mutants of *argR2*, *mntR*, *atlR*, *rbf*, *sarU*, *xdrA*, and *rex* were grown under standard conditions in TSB. Data are from three biological replicates with error bars showing SDs. Download FIG S2, PDF file, 0.1 MB. Copyright © 2019 Gimza et al. 2019 Gimza et al. This content is distributed under the terms of the Creative Commons Attribution 4.0 International license.

**FIG 6:**
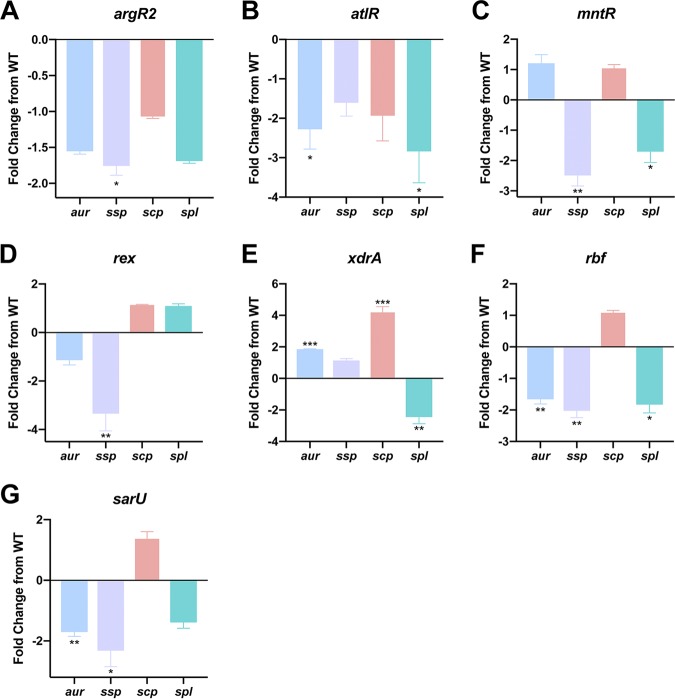
Differential control of individual protease loci by a secondary network of regulatory factors. qRT-qPCR was performed to determine transcript levels for *aur*, *ssp*, *scp*, and *spl* in the regulator mutants. The strains used were WT USA300 HOU and mutants of *argR2* (A), *atlR* (B), *mntR* (C), *rex* (D), *xdrA* (E), *rbf* (F), and *sarU* (G). RNA was isolated from three independent cultures. The 16S rRNA gene was used as an internal control. Fold change from WT was determined using the 2^−ΔΔ^*^CT^* method. Student’s *t* tests were used to determine statistical significance. *, *P* < 0.05; **, *P* < 0.01; ***, *P* < 0.001 relative to the wild-type strain. Error bars are SDs.

Our next step was to determine if the changes observed in the novel regulatory mutants were driven by changes at the level of transcription. Thus, qRT-PCR analysis for each protease loci was performed for the wild-type and regulator mutant strains during postexponential phase, with the exception of the *argR2* mutant, which appears to most notably alter proteolysis at 3 h of growth; thus, this time point was used for this strain. When studying changes in the *argR2* mutant, a 1.6-fold decrease in *aur*, 1.8-fold increase in *sspA*, and 1.7-fold increase in *spl* transcripts were observed (Fig. 6A), along with no change in *scpA* transcription. Next, with the mutant of *atlR*, we observed a significant 2-fold decrease in *aur* and a 2.2-fold decrease in *spl* transcripts (Fig. 6B), whereas with *scpA* and *sspA*, no changes in transcript levels were noted. For the *mntR* mutant, we observed a significant 2.5-fold decrease in *sspA* and 1.7-fold decrease in *spl* transcript levels (Fig. 6C), with no changes detected for *aur* and *scpA*. In the context of *rex*, a significant 3.3-fold decrease was seen with *sspA* transcript levels, while there were no changes in transcription for the other protease loci in this mutant (Fig. 6D). Following this, we investigated the *xdrA* mutant, in which we observed a significant 1.9-fold increase for *aur* and 4.2-fold increase in *scpA* transcript levels (Fig. 6E); however, with *spl*, we observed a significant 2.4-fold decrease in expression. When studying the *rbf* mutant, there was a significant 1.7-fold decrease for *aur*, 2-fold decrease for *sspA*, and 1.8-fold decrease for *spl* transcript levels (Fig. 6F), along with no changes for *scpA* transcription. Lastly, for the *sarU* mutant, we observed a significant 2.3-fold decrease for *sspA* and 1.7-fold decrease for *aur* transcript levels (Fig. 6G), while no changes were noted for *scpA* and *spl* transcription. Collectively, almost all of the regulators solely activate protease transcription, with the exception of XdrA, which differentially regulates protease loci in opposing fashions, akin to that observed with Rot and SarS.

### Determining the pathway of control for the novel protease regulators.

**FIG 7:**
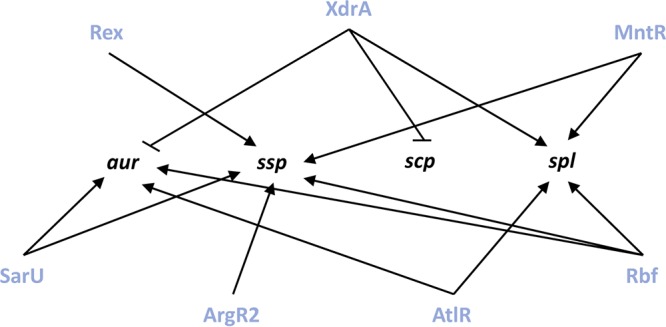
Novel regulatory network controlling expression of extracellular proteases. Shown are transcriptional regulation events for the seven novel protease regulators on the four individual protease loci. Bars indicate repression, and arrows indicate activation.

In the work described above, we identified 14 new regulatory pathways for secreted protease transcription. These data allow us to construct a map of protease regulation for these factors, detailing specific effects on individual protease loci (Fig. 7). To delineate the pathway by which these regulators exert their effects, we next assessed their impact on the primary regulators of protease expression considered previously. As such, qRT-PCR analysis was performed on the seven novel protease regulator mutants for *sarA*, *codY*, *rot*, *sarS*, *saeR*, *mgrA*, and *sarR* at the respective time points in which their protease transcripts were previously assessed. SarA, SarR, MgrA, and CodY are able to regulate protease production by direct action, but can also act via control of the Agr quorum sensing system (26, 66–72). Agr in turn activates secreted protease production during postexponential phase by inhibiting translation of the negative regulator Rot (73–75). As such, for completeness, we also included analysis of the *agr* operon in these studies.

**FIG 8:**
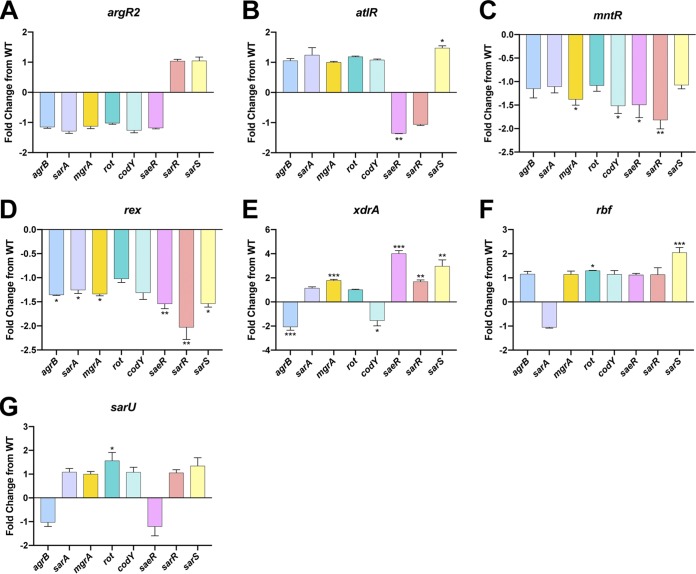
Determining the pathway of control for the novel protease regulators. qRT-PCR was performed to determine transcript levels for *agrB*, *sarA*, *mgrA*, *rot*, *codY*, *saeR*, *sarR*, and *sarS* in the regulator mutants. The strains used were WT USA300 HOU and mutants of *argR2* (A), *atlR* (B), *mntR* (C), *rex* (D), *xdrA* (E), *rbf* (F), and *sarU* (G). RNA was isolated from three independent cultures. The 16S rRNA gene was used as an internal control. Fold change from the WT was determined using the 2^−ΔΔ^*^CT^* method. Student’s *t* tests were used to determine statistical significance. *, *P* < 0.05; **, *P* < 0.01; ***, *P* < 0.001 relative to the wild-type strain. Error bars are SDs.

When data for the *argR2* mutant were analyzed, we found no significant changes in expression for any of the primary protease regulators (Fig. 8A). As such, the changes in *ssp* transcript levels in the *argR2* mutant are either the result of direct action by ArgR2 or are mediated by an as yet unknown circuit. When assessing the *atlR* mutant, a significant 1.4-fold decrease in *saeR* and a 1.5-fold increase in *sarS* transcripts were observed (Fig. 8B). The decrease in *saeR* could explain the observed decrease in *spl* expression, as SaeR was shown by ourselves and others to activate *spl* transcription (24, 39). In addition, the increase in *sarS* expression could explain the decrease in both *aur* and *spl* transcripts, as SarS was shown in this study to repress transcription of *spl* and was shown here and elsewhere to repress *aur* expression (25).

Next, with the *mntR* mutant, we observed a significant 1.4-fold decrease in *mgrA*, 1.5-fold decrease in *codY*, 1.5-fold decrease in *saeR*, and 1.8-fold decrease in *sarR* transcript levels (Fig. 8C). With regard to the decrease in *ssp* and *spl* transcripts, these changes cannot be explained by the decrease in transcription for *codY*, as we show that CodY represses both of these loci. The decrease in the *saeR* transcript, however, could result in a decrease in *spl* transcription, as it has been shown by ourselves and others to be an activator of this operon (24, 39). Furthermore, the decrease in *mgrA* and *sarR* transcripts could lead to a decrease in *ssp* and *spl* expression, as we confirm the work of others demonstrating that MgrA activates expression for both proteases (25, 34) while newly identifying SarR as acting in a similar fashion.

When exploring the influence of Rex, we observed a significant 1.4-fold decrease in *agrB*, 1.3-fold decrease in *sarA*, 1.3-fold decrease in *mgrA*, 1.5-fold decrease in *saeR*, 2-fold decrease in *sarR*, and 1.5-fold decrease in *sarS* transcript levels (Fig. 8D). The changes in *sarA*, *saeR*, and *sarS* cannot explain the decrease we observed for the *ssp* transcript, because as shown by ourselves and others, both are repressors of *ssp* (9, 10, 25). However, as we and others have shown that MgrA, SarR, and Agr are activators of *ssp* transcription (9, 21, 25), decreases in their expression could explain our data. When assessing the *xdrA* mutant, a significant 2.1-fold decrease in *agrB* and 1.5-fold decrease in *codY* transcript levels were observed (Fig. 8E). Additionally, a significant 1.8-fold increase in *mgrA*, 4-fold increase in *saeR*, 1.7-fold increase in *sarR*, and 3-fold increase in *sarS* transcripts were observed. The increase in *mgrA* transcript could explain the increase in *aur* expression as MgrA has been shown here and by others to activate its transcription (25, 34). Next, as we showed SaeR, SarR, and SarS are activators of *scp* expression, increases in the transcription of each could result in enhanced *scp* transcript abundance. Additionally, the decrease in *codY* expression could explain the increase in transcript for *aur* and *scp*, as we showed CodY is a repressor of both. Lastly, the decrease in *spl* transcript levels in the *xdrA* mutant could be explained by either the increase in *sarS* or by the decrease in *agrB* expression, as we show that SarS is a repressor of this locus, while it is well known that Agr is an activator of *spl* transcription (10). Next, with the *rbf* mutant, we observed a significant 1.3-fold increase in *rot* transcription as well as a 2.1-fold increase for *sarS* (Fig. 8F). The decrease in *aur* and *ssp* transcript levels observed in the *rbf* mutant could be explained by the increase in *sarS* expression, as it was shown by ourselves and others to be a repressor for both loci (25). Furthermore, we show SarS is a repressor of *spl*, and as such, the increase in *sarS* could have resulted in the decrease in the *spl* transcript. In addition, Rot was shown herein, and by others, to be a repressor for *aur* and *ssp*; therefore, the increase in *rot* transcription could result in the decrease of *aur* and *ssp* expression (23). Lastly, with the *sarU* mutant, we observed a significant 1.6-fold increase in *rot* transcription (Fig. 8G). In the *sarU* mutant, the decrease in *aur* and *ssp* transcription could be explained by the increase in *rot* transcription, as it has been shown by ourselves and others to be a repressor of both (23).

### Integrating the novel secondary protease regulators into the global picture of protease control.

**FIG 9:**
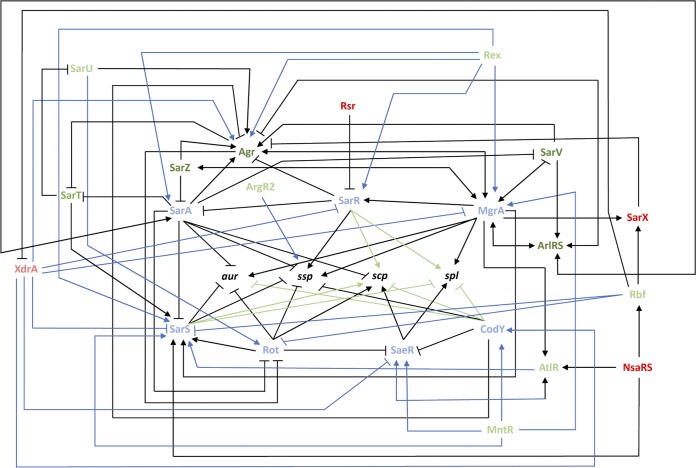
Mapping the global network of extracellular protease regulation in Staphylococcus aureus. The seven primary regulators of protease expression are shown in blue, while factors known to, in turn, regulate their expression are shown in dark green (activators) or dark red (repressors). The novel regulators identified in this study are shown in light green (activators) or light red (repressors). New regulatory pathways identified herein between the primary regulators and the protease loci are shown in green. New regulatory pathways identified herein between the primary regulators and the novel regulators are shown in blue.

Using the findings from this study, along with existing knowledge, we put forward a comprehensive map of secreted protease regulation (Fig. 9). With this knowledge, we are able to identify specific regulatory pathways connecting our novel protease effectors with the major protease regulators. Specifically, with regard to Rbf, it is possible that its repressive effect on *sarS* transcription is through Rot, as it was previously shown to activate *sarS* transcription (35, 71) and *rot* transcription is increased in the *rbf* mutant. Next, with MntR, its positive effect on *sarR* transcription is likely occurring through MgrA, as it was previously shown that MgrA activates *sarR* transcription (34) and *mgrA* expression is decreased in the *mntR* mutant. As for Rex, its activation of *sarR* transcription could be occurring through MgrA, as it has been shown that MgrA activates *sarR* (35, 71) and *mgrA* transcription is decreased in the absence of *rex*. Lastly, with XrdA, it is possible that its represses *saeR* via CodY, as it has been shown that CodY represses *saeR* transcription (76, 77) and *codY* transcription is decreased in the *xdrA* mutant. Additionally, the negative effect of XdrA on *sarR* and *sarS* transcription could be occurring via MgrA, as it was previously shown that MgrA activates *sarR* and *sarS* transcription (34, 71) and *mgrA* transcription is increased in the *xdrA* mutant. Finally, the activation of *agr* by XdrA could by occurring via the MgrA-SarR pathway, as SarR has been shown to repress *agr* transcription (68) and, as already noted, *sarR* transcription is increased in the *xdrA* mutant.

### Concluding remarks.

In this study, we set out to completely characterize the locus-specific effects of regulatory factors on secreted protease expression. In so doing, we have identified an abundance of novel regulatory nodes controlling their production and present a comprehensive regulatory circuit that emphasizes the complexity of protease regulation (Fig. 9). When one compares this regulatory overview with the literature on virulence factor control in S. aureus, it becomes clear that the expansive and complex regulatory circuits that exist to oversee secreted protease expression rivals that of alpha-toxin and protein A, which are arguably some of the most important virulence-affecting entities produced by this organism (35, 65, 78–85). Indeed, we suggest that the existence of such a broad network of control speaks to the importance of the secreted proteases to S. aureus physiology and pathogenic potential. We also contend that there is a clear and obvious need for such a network, so as to limit or enhance the abundance (and thus activity) of these enzymes. The rationale for this is that a primary function of these enzymes is to control the progression of infection by selectively modulating the stability of individual virulence factors produced by the cell (19). Thus, in this context, it makes sense that a network of control exists to selectively titrate in or out a given protease (and thus its activity), so as to specifically influence the abundance (or lack thereof) of an individual virulence factor(s). This would then facilitate the selective and niche-specific pathogenic behaviors of S. aureus and provide a basis for control of the broad and varied infection types that is the hallmark of this organism’s disease-causing nature. In addition to this, there is abundant evidence in the literature implicating the secreted proteases as facilitating the infectious process by attacking the host and cleaving host proteins. It is thus in line with the above hypothesis that tightly controlling protease activity, by selectively limiting or enhancing their activity in specific niches, is to the advantage of S. aureus and its highly effective and efficient infectious process.

## MATERIALS AND METHODS

### Media and growth conditions.

All cultures were grown overnight at 37°C with shaking at 250 rpm in 5 ml of either tryptic soy broth (TSB) or lysogeny broth (LB). When required, antibiotics were added at the following concentrations: for Escherichia coli, 100 μg ml^−1^ ampicillin, 12.5 μg ml^−1^ tetracycline; for S. aureus, 5 μg ml^−1^ tetracycline, 5 μg ml^−1^ erythromycin, 25 μg ml^−1^ lincomycin, and 2.5 μg ml^−1^ chloramphenicol. To obtain synchronous cultures, overnight S. aureus cultures were diluted 1:100 into 5 ml of fresh medium and grown for 3 h before being standardized to an optical density at 600 nm (OD_600_) of 0.05 in 100 ml of fresh TSB. When assessing growth, OD_600_ was measured hourly using a Synergy 2 plate reader (Bio-Tek).

### Bacterial strains.

All bacterial strains and plasmids used in this study are listed in Table 1. Transposon mutants for all available transcriptional regulators in S. aureus USA300 JE2 were obtained from the Nebraska Transposon Mutant Library (NTML). Those subjected to further study were transduced into USA300 Houston, as described by us previously (86), using ϕ11. The construction of an *mgrA* mutant in S. aureus Becker was previously described (87). This mutation was transduced into USA300 Houston using ϕ85.

**TABLE 1:** Strains and plasmids used in this study

Strain or plasmid	Description^*a*^	Reference or source
Strains		
E. coli		
DH5α	Cloning strain	92
S. aureus		
RN4220	Restriction-deficient strain	Lab stock
USA300 HOU	USA300 HOU MRSA isolate	58
BDG2625	USA300 HOU *codY*::Tn::*erm* Δ*codY*	This study
BDG2623	USA300 HOU *sarS*::Tn::*erm* Δ*sarS*	This study
BDG2621	USA300 HOU *sarA*::Tn::*erm* Δ*sarA*	This study
BDG2624	USA300 HOU *rot*::Tn::*erm* Δ*rot*	This study
BDG2622	USA300 HOU *saeR*::Tn::*erm* Δ*saeR*	This study
CYL1040	Becker *mgrA*::*cm* Δ*mgrA*	87
BDG2626	USA300 HOU *mgrA*::*cm* Δ*mgrA*	This study
BDG2479	USA300 HOU *sarR*::*tet* Δ*sarR*	This study
BDG2331	USA300 HOU *sarU*::Tn::*erm* Δ*sarU*	This study
BDG2333	USA300 HOU *rex*::Tn::*erm* Δ*rex*	This study
BDG2329	USA300 HOU *rbf*::Tn::*erm* Δ*rbf*	This study
BDG2334	USA300 HOU *argR2*::Tn::*erm* Δ*argR2*	This study
BDG2336	USA300 HOU *atlR*::Tn::*erm* Δ*atlR*	This study
BDG2328	USA300 HOU *mntR*::Tn::*erm* Δ*mntR*	This study
BDG2332	USA300 HOU *xdrA*::Tn::*erm* Δ*xdrA*	This study
LES55	SH1000 *sigS*::*tet* Δ*sigS*	89
Plasmids		
pJB38	Plasmid to create mutants in S. aureus	88
pBDG01	pJB38 construct for *sarR* mutation, Amp^r^ CM^r^	This study

a Erm, erythromycin; CM, chloramphenicol; Tet, tetracycline; Amp, ampicillin.

### Construction of a *sarR* mutant strain.

A tetracycline-marked disruption of *sarR* was generated using pJB38, as described by Bose et al. (88). Regions up- and downstream of *sarR*, including portions of the 5′ and 3′ ends of the coding gene, were amplified via PCR using primers OL4208/OL4209 and OL4210/OL4211. A tetracycline resistance cassette was amplified using OL4299/OL4300 from a SH1000 *sigS*::*tet* mutant (89). Using MluI sites, the tetracycline cassette was ligated between the upstream and downstream fragments of *sarR* and ligated directly into pJB38 using EcoRI and KpnI sites. Using the established protocol, the majority of *sarR* was deleted in USA300 Houston using allelic replacement (88). Strains were confirmed by PCR and sequencing (Eurofins Genomics) using primers OL4577/OL4578, which amplify across the deleted region where the tetracycline cassette was inserted.

### Quantitative real-time PCR analysis.

To quantify expression changes for target genes (primers are listed in Table S3 in the supplemental material), quantitative real-time PCR (qRT-PCR) was performed, as described by us previously (90). All targets were normalized using 16S rRNA expression, and fold change from the wild-type was determined using the threshold cycle (2^−ΔΔ^*^CT^*) method (91). All graphical representations of fold changes are relative to the wild-type, ±1.

10.1128/mSphere.00676-19.5 TABLE S3 Primers used in this study. *^a^* Restriction sites present in primers are denoted by underlining. *^b^* KO, knockout. Download Table S3, PDF file, 0.03 MB. Copyright © 2019 Gimza et al. 2019 Gimza et al. This content is distributed under the terms of the Creative Commons Attribution 4.0 International license.

### Zymography.

Strains grown for 15 h overnight were adjusted to equal optical densities and pelleted. When assessing proteolytic activity over time, synchronized cultures were grown to exponential phase and standardized to an OD_600_ of 0.05 in 100 ml of TSB. At the desired time points, cells were pelleted. Thereafter, for all samples, 2 ml of supernatant was processed through an Amicon Ultra 3K centrifugal filter for 60 min at 4,000 × *g*. Concentrated supernatants were recovered by removing filtrate collection tubes, inverting filter devices, and spinning again for 2 min at 1,000 × *g*. Equal volumes of Laemmli loading buffer were added to the concentrated supernatants and incubated for 30 min at 37°C. Next, 20 μl of each sample was loaded onto preprepared SDS-PAGE gels containing 0.1% gelatin and run until the dye front reached the edge of the plates. Gels were washed twice using 2.5% Triton X-100 at room temperature. Following a rinse with distilled water (dH_2_O), developing buffer (0.2 M Tris, 5 mM CaCl_2_, 1 mM dithiothreitol [DTT], pH 7.6) was added and gels were incubated overnight at 37°C static. After incubation, gels were rinsed with dH_2_O and covered with 0.1% amido black for 1 h. Once gels were stained, destain 1 (30% methanol, 10% acetic acid) was added for 5 to 10 min, replaced with destain 2 (10% acetic acid) until bands became clear, and then replaced with destain 3 (1% acetic acid) for storage. Changes in band intensity were quantified using ImageJ software.
