# Supplementary material for: Mapping the Global Network of Extracellular Protease Regulation in Staphylococcus aureus
Source: mSphere. 2019 Oct 23;4(5):e00676-19. doi: 10.1128/mSphere.00676-19 (PMC6811363; doi:10.1128/mSphere.00676-19)
Supplement: TABLE S1 [file mSphere.00676-19-st001.pdf]

Gene ID[a]	NE#[b]	Name[c]	Family[d]
SAUSA300_0066	1233	ArgR2	ArgR
SAUSA300_0093	505	N/A	LyTR
SAUSA300_0217	49	N/A	TCS-RR
SAUSA300_0621	431	MntR	FeoA
SAUSA300_0653	872	Rbf	AraC/XylS
SAUSA300_0658	1557	CcpE	LysR
SAUSA300_0691	1622	SaeR	TCS-RR
SAUSA300_0954	1543	AtlR	MarR/SlyA
SAUSA300_1019	456	N/A	Xre
SAUSA300_1455	1566	N/A	AraC/XylS
SAUSA300_1969	523	N/A	Xre
SAUSA300_1992	1391	AgrA	TCS-RR
SAUSA300_1999	1158	Rex	Rex
SAUSA300_2050	1971	N/A	TenA
SAUSA300_2106	837	MtlR	BglG
SAUSA300_2156	436	LacR	GntR
SAUSA300_2218	1941	SarV	Sar
SAUSA300_2248	1532	N/A	AraC/XylS
SAUSA300_2279	1511	HutR	LysR
SAUSA300_2310	671	N/A	LyTR
SAUSA300_2437	514	SarT	Sar
SAUSA300_2438	1471	SarU	Sar
SAUSA300_2445	1534	N/A	MerR
SAUSA300_2459	1560	N/A	MarR/SlyA
SAUSA300_2509	1295	N/A	TetR
SAUSA300_2547	1201	N/A	N/A
