# Supplementary material for: Mapping the Global Network of Extracellular Protease Regulation in Staphylococcus aureus
Source: mSphere. 2019 Oct 23;4(5):e00676-19. doi: 10.1128/mSphere.00676-19 (PMC6811363; doi:10.1128/mSphere.00676-19)
Supplement: TABLE S2 [file mSphere.00676-19-st002.pdf]

Gene ID[a]	NE#[b]	Name[c]	Family[d]
SAUSA300_0104	78	N/A	AraC/XylS
SAUSA300_0114	165	SarS	Sar
SAUSA300_0195	1659	RpiRB	RpiR
SAUSA300_0373	1226	N/A	Xre
SAUSA300_0605	1193	SarA	Sar
SAUSA300_0645	481	GraR	TCS-RR
SAUSA300_0654	296	SarX	Sar
SAUSA300_0804	1950	N/A	Xre
SAUSA300_0998	1362	N/A	Xre
SAUSA300_1148	1555	CodY	CodY
SAUSA300_1469	1895	ArgR3	ArgR
SAUSA300_1708	386	Rot	Sar
SAUSA300_1797	1389	XdrA	Xre
SAUSA300_1888	1955	HisR	Trp
SAUSA300_2022	1109	SigB	Sigma Factor
SAUSA300_2247	210	SarY	Sar
SAUSA300_2322	1064	N/A	TetR
SAUSA300_2331	567	SarZ	Sar
SAUSA300_2559	1643	NsaR	TCS-RR
