# Supplementary figures and images for: Mapping the Global Network of Extracellular Protease Regulation in Staphylococcus aureus
Source: mSphere. 2019 Oct 23;4(5):e00676-19. doi: 10.1128/mSphere.00676-19 (PMC6811363; doi:10.1128/mSphere.00676-19)

A

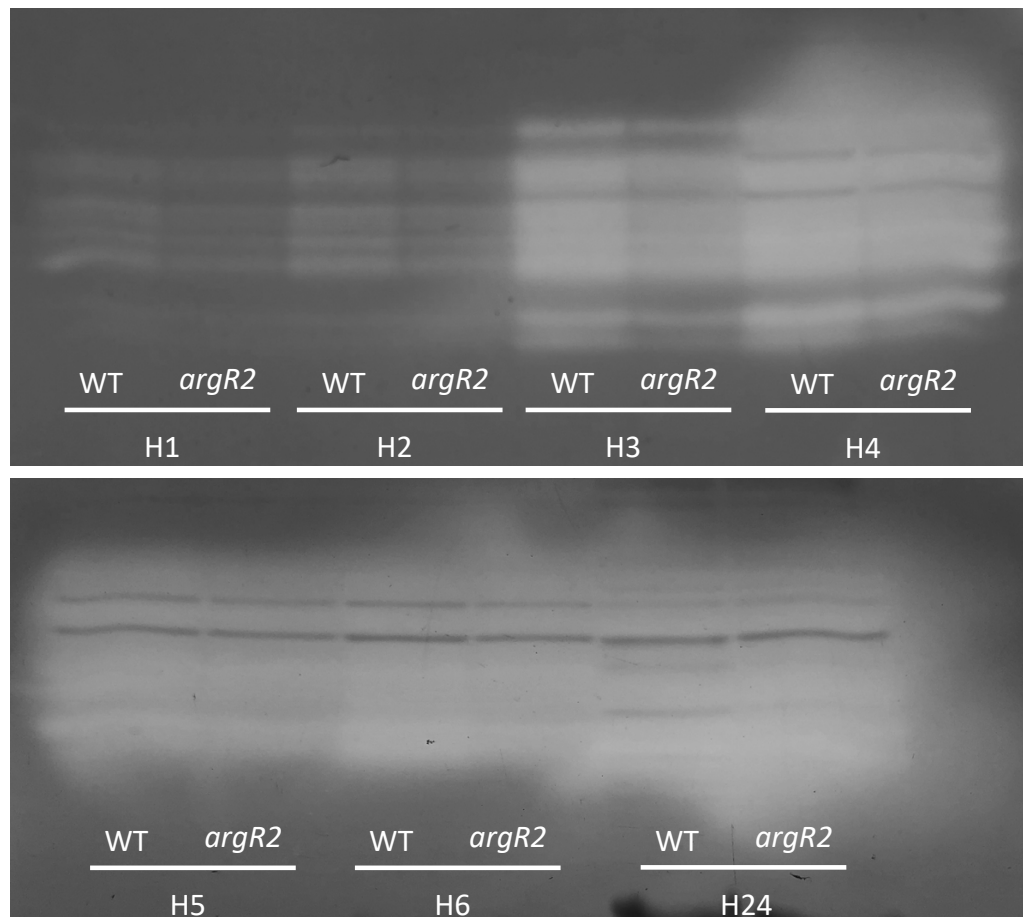

B

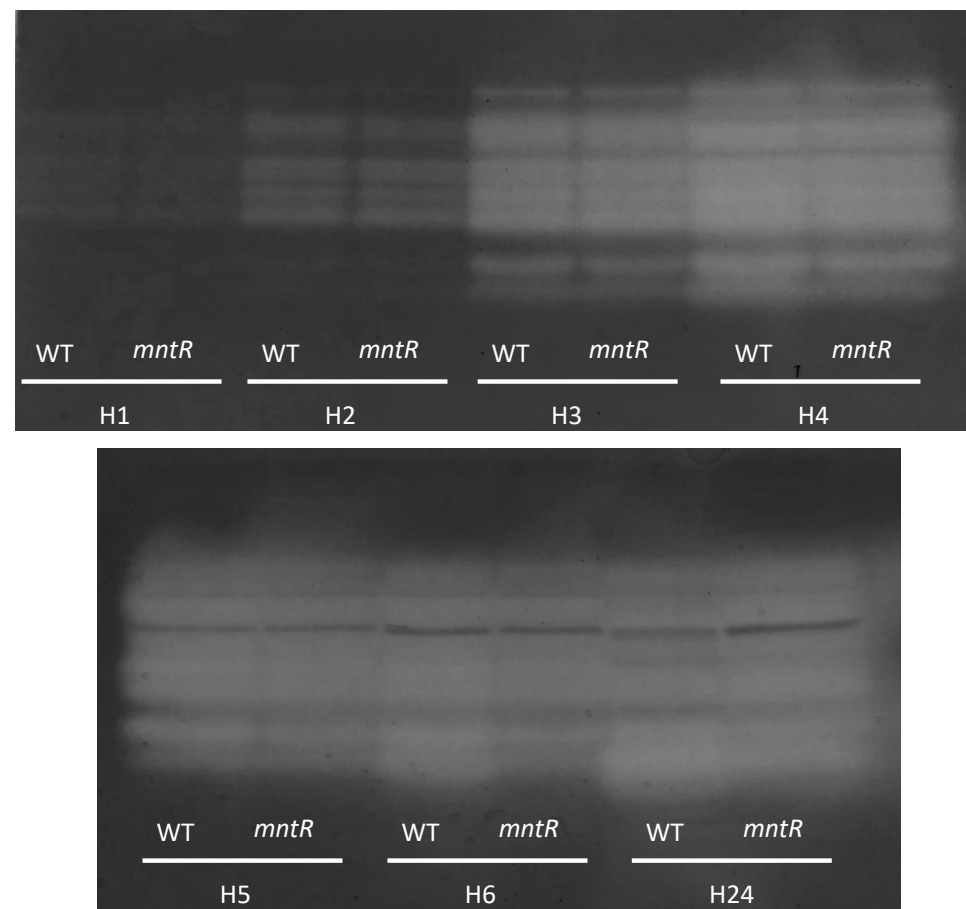

C

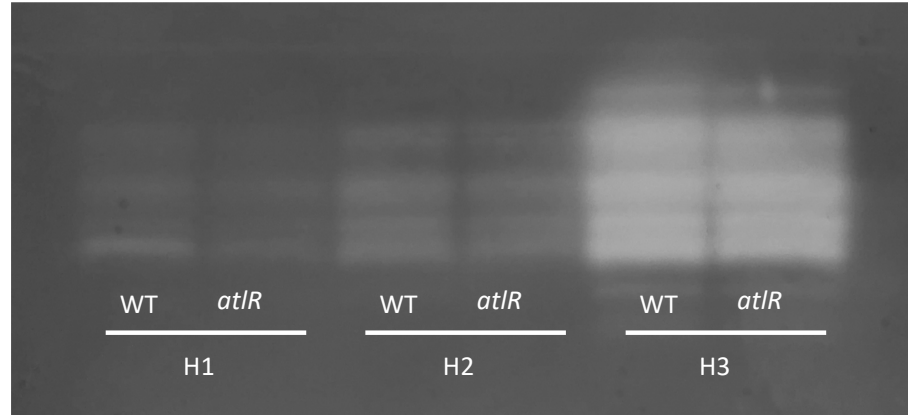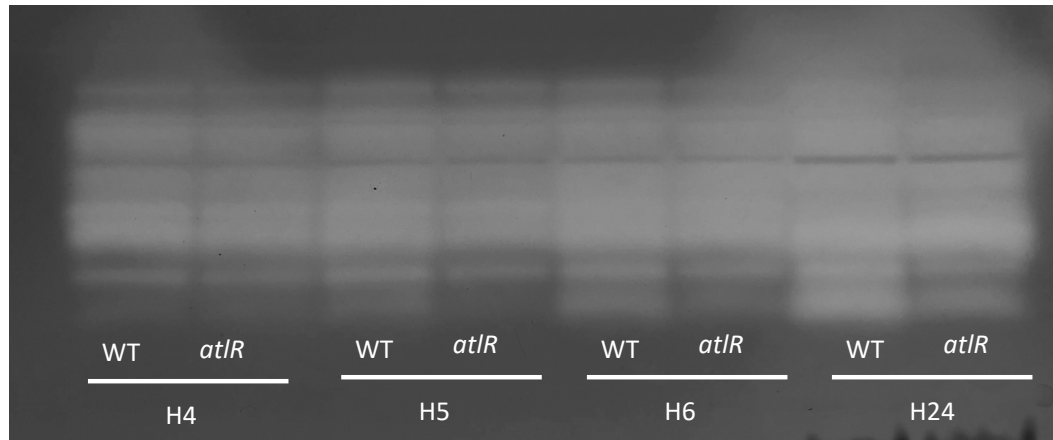

D

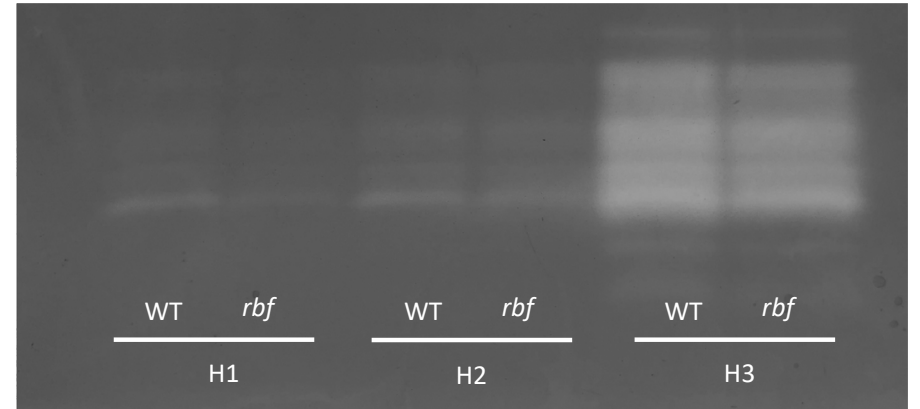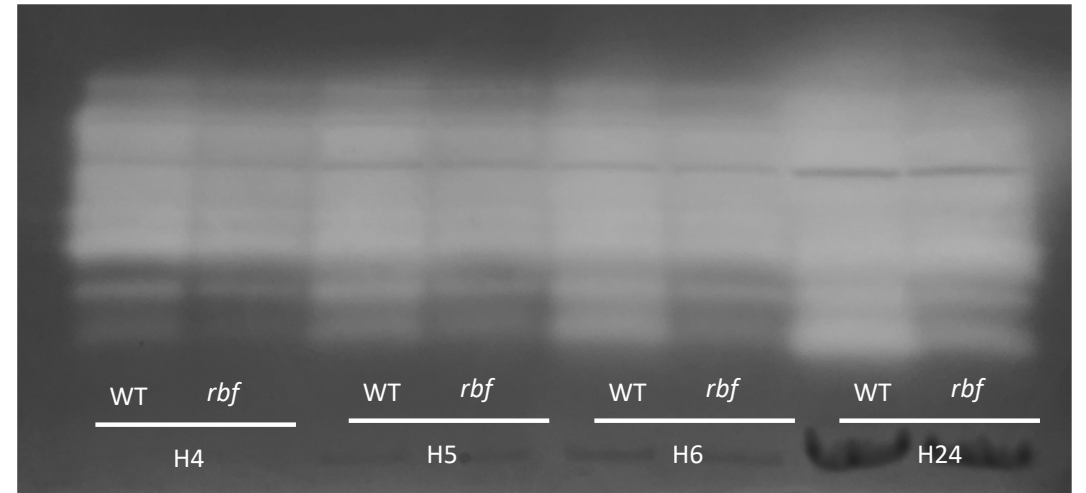

E

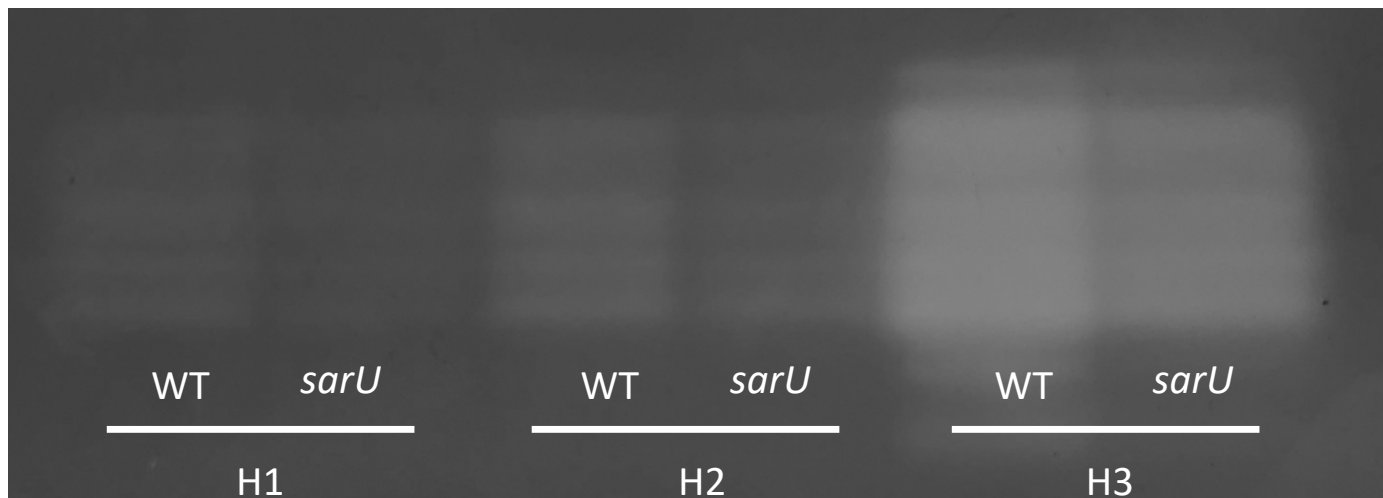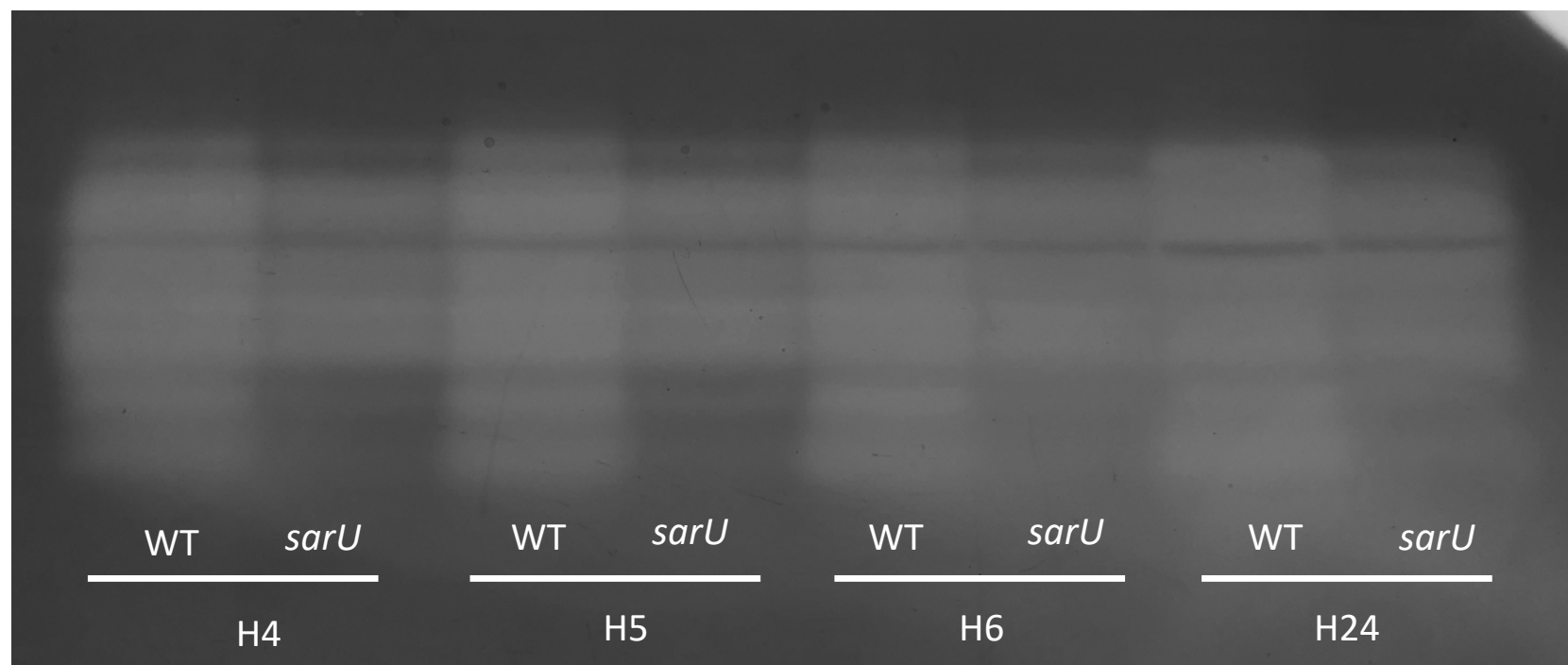

F

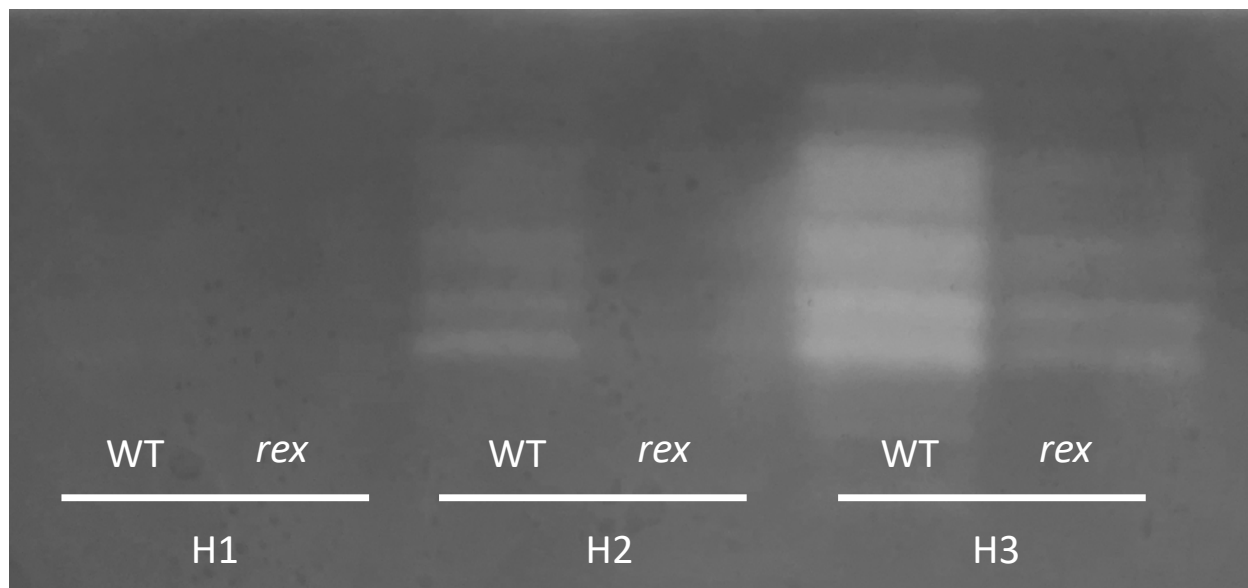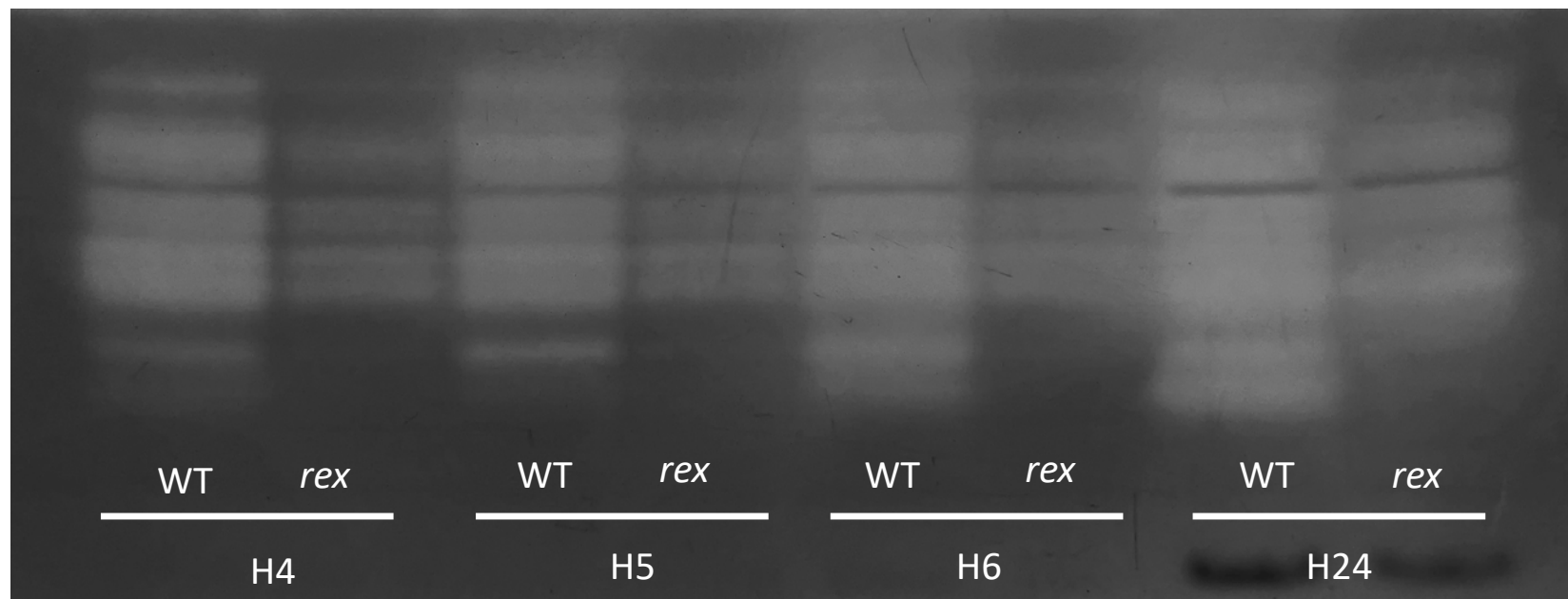

G

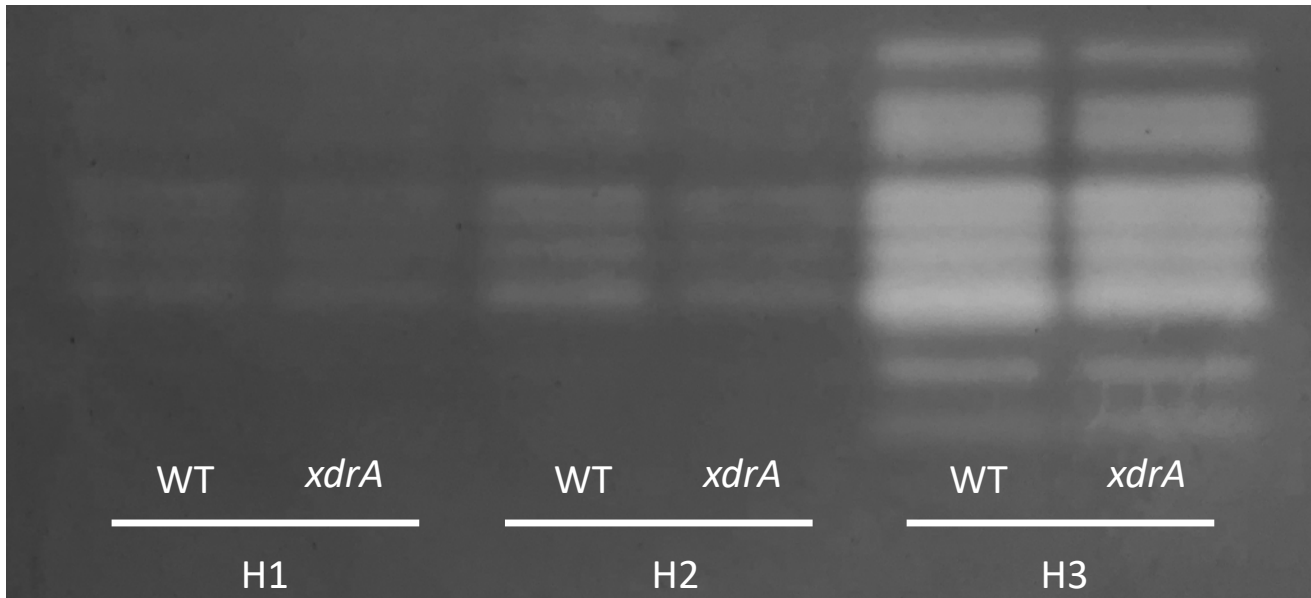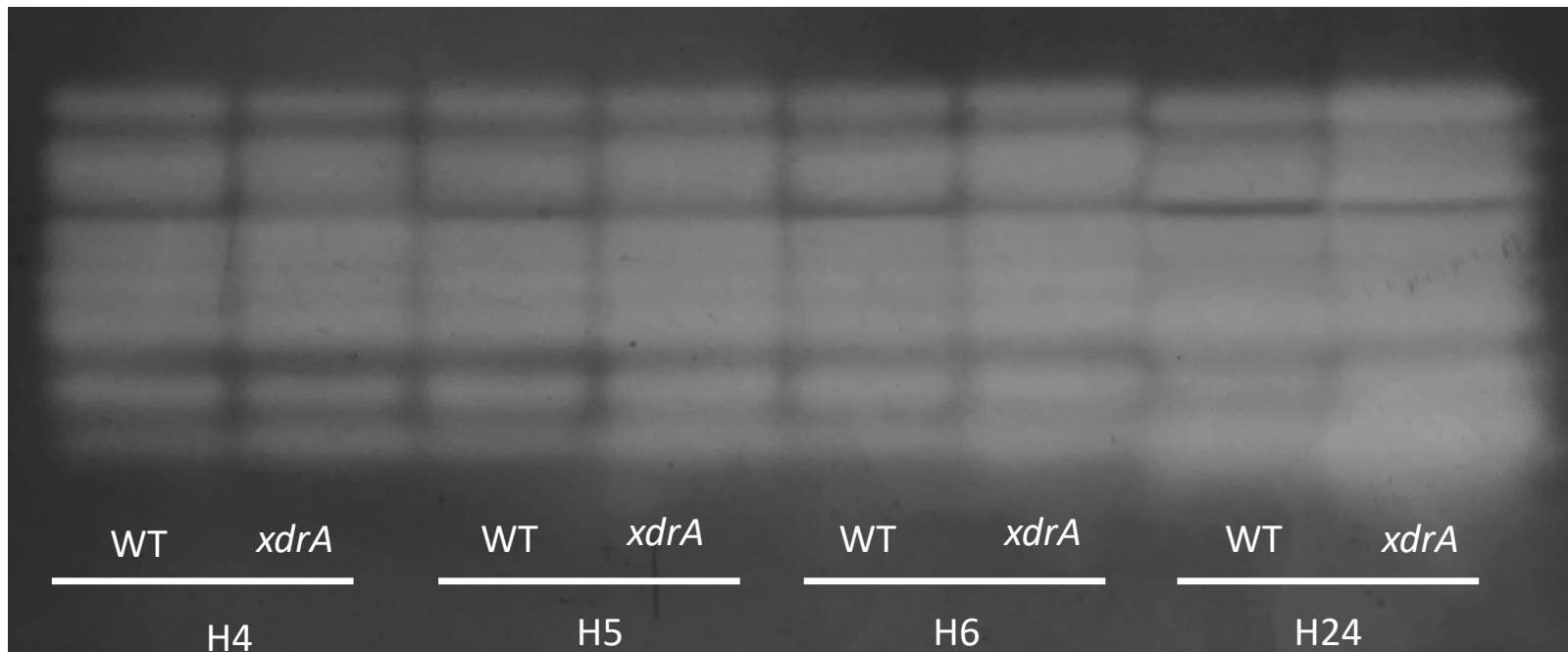

Supplement: FIG S1 [file mSphere.00676-19-sf001.pdf]

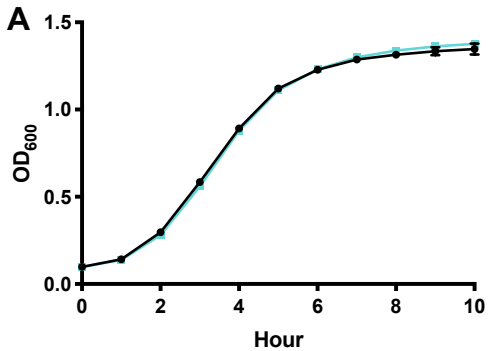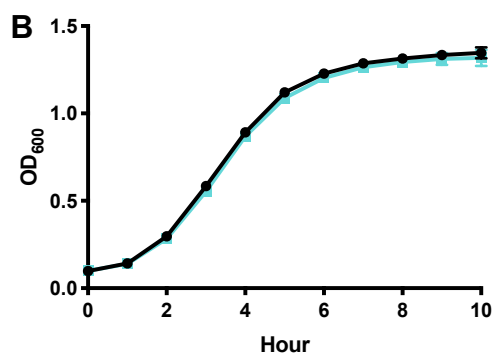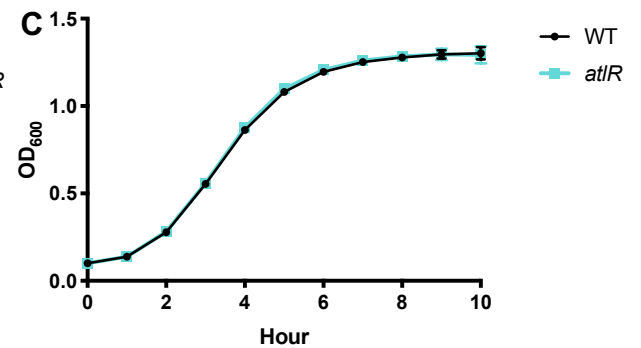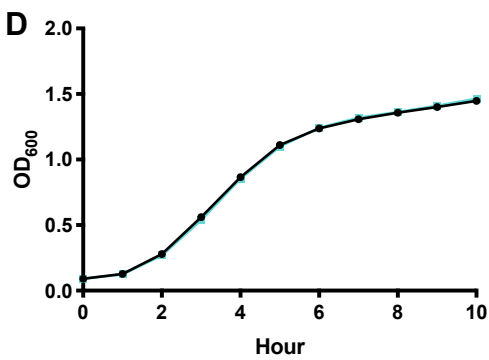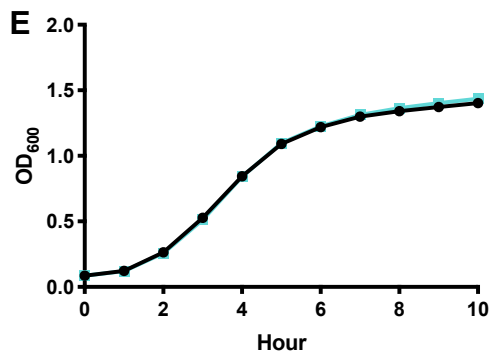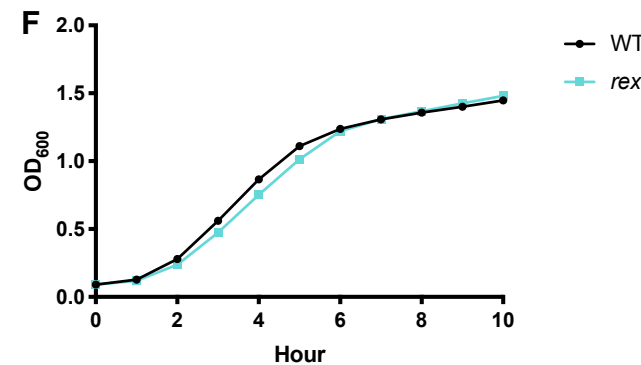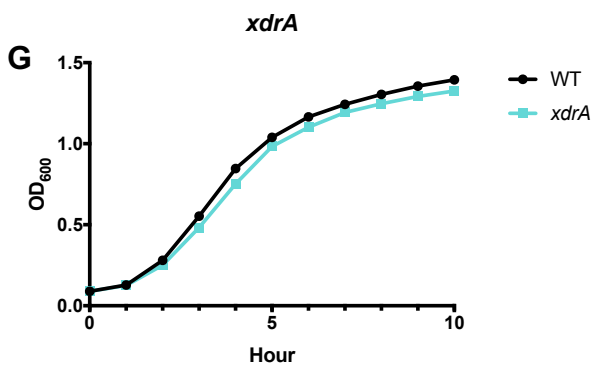

Supplement: FIG S2 [file mSphere.00676-19-sf002.pdf]
