# Supplementary material for: Mapping the Global Network of Extracellular Protease Regulation in Staphylococcus aureus
Source: mSphere. 2019 Oct 23;4(5):e00676-19. doi: 10.1128/mSphere.00676-19 (PMC6811363; doi:10.1128/mSphere.00676-19)
Supplement: TABLE S3 [file mSphere.00676-19-st003.pdf]

Primer	Sequence[a]	Description[b]
OL2123	GAGTTGTTATCAATGGTCAC	*sarA* qPCR
OL2124	ACTGCTTTAACAACTTGTGG	*sarA* qPCR
OL2536	AGTTTGCCACGTATCTT	*agrB* qPCR
OL2537	TTAGCTAAGACCTGCATC	*agrB* qPCR
OL3747	CGTAGATGCAAATTATTACG	*aur* qPCR
OL3748	CGTTAATGAAACAATTGGAC	*aur* qPCR
OL3749	GTAGTTGTAGGTAAAGATACTC	*sspA* qPCR
OL3750	CCATTTGGATAATTGTCTTG	*sspA* qPCR
OL3751	GAACACTGAAGGTAATATCG	*scpA* qPCR
OL3752	GGAGAAACATTGATTGTGTA	*scpA* qPCR
OL2491	GGATTTGTAGTTGGAAAGA	*splB* qPCR
OL2492	CTATTTGGATGTGCAGTAA	*splB* qPCR
OL4047	GCTCAAAGACAAGTTAATCGCTAC	*mgrA* qPCR
OL4048	CGTTTACAGGAGATTCATCCCA	*mgrA* qPCR
OL4034	CAGCGAGATTGAAAGCGAATAC	*rot* qPCR
OL4035	CTGTCCATTTCTTTAAGCGTCATAG	*rot* qPCR
OL4011	CTAGGTGAATATGCTGCTACAG	*codY* qPCR
OL4012	CCATTGTAATAGCAGCTTTATCG	*codY* qPCR
OL3116	ACCACAATAACTCAAATTCCTTAATACG	*saeR* qPCR
OL3117	GTTGAACAACTGTCGTTTGATGA	*saeR* qPCR
OL4445	AACTGTTCTTTCGTCTTGTAAACT	*sarR* qPCR
OL4446	TGCTCAGAGTTCAAACCTTACT	*sarR* qPCR
OL2495	AACAATCGGATTTAGTACAG	*sarS* qPCR
OL2496	GTAAGTATTACGCTCATCAA	*sarS* qPCR
OL1184	AGCCGACCTGAGAGGGTGA	*16s* qPCR
OL1185	TCTGGACCGTGTCTCAGTTCC	*16s* qPCR
OL4208	ATGGAATTCCTAAATCAATCTACCATTCCTACAT	*sarR* KO A fragment
OL4209	ATGACGCGTCAGTTATTGTTTATGTTACAGATACAC	*sarR* KO A fragment
OL4210	ATGACGCGTGAAATGTTGCGTTGACTAAATC	*sarR* KO B fragment
OL4211	ATGGGTACCGTCACTATGCTTATTCAAGCAT	*sarR* KO B fragment
OL4299	ATGACGCGTGAGTAATGCTAACATAGC	Tetracycline cassette
OL4300	ATGACGCGTCCCAAAGTTGATCCCTTAACG	Tetracycline cassette
OL4577	GACTAGTGTACCTTGTTTCAAGC	*sarR* KO screening
OL4578	TTGCTACAACAAGATGTGCATC	*sarR* KO screening
